# Genome-wide TCP transcription factors analysis provides insight into their new functions in seasonal and diurnal growth rhythm in *Pinus tabuliformis*

**DOI:** 10.1186/s12870-022-03554-4

**Published:** 2022-04-02

**Authors:** Yu-meng Nie, Fang-xu Han, Jing-jing Ma, Xi Chen, Yi-tong Song, Shi-Hui Niu, Harry X. Wu

**Affiliations:** 1 Beijing Advanced Innovation Center for Tree Breeding by Molecular Design, National Engineering Laboratory for Tree Breeding, College of Biological Sciences and Technology, Beijing Forestry University, 100083 Beijing, PR China; 2 Umeå Plant Science Centre, Department of Forest Genetics and Plant Physiology, Swedish University of Agricultural Sciences, Linnaeus väg 6, SE-901 83 Umeå, Sweden

**Keywords:** *TCP*, Gene family, *Pinus tabuliformis*, Seasonal, Diurnal, Oscillation

## Abstract

**Background:**

*Pinus tabuliformis* adapts to cold climate with dry winter in northern China, serving as important commercial tree species. The TEOSINTE BRANCHED 1, CYCLOIDEA, and PROLIFERATING CELL FACTOR family(TCP)transcription factors were found to play a role in the circadian clock system in *Arabidopsis*. However, the role of TCP transcription factors in *P. tabuliformis* remains little understood.

**Results:**

In the present study, 43 *TCP* genes were identified from *P. tabuliformis* genome database. Based on the phylogeny tree and sequence similarity, the 43 *TCP* genes were classified into four groups. The motif results showed that different subfamilies indeed contained different motifs. Clade II genes contain motif 1, clade I genes contain motif 1, 8, 10 and clade III and IV contain more motifs, which is consistent with our grouping results. The structural analysis of *PtTCP* genes showed that most *PtTCPs* lacked introns. The distribution of clade I and clade II on the chromosome is relatively scattered, while clade III and clade IV is relatively concentrated. Co-expression network indicated that *PtTCP2*, *PtTCP12*, *PtTCP36*, *PtTCP37*, *PtTCP38*, *PtTCP41* and *PtTCP43* were co-expressed with clock genes in annual cycle and their annual cycle expression profiles both showed obvious seasonal oscillations. *PtTCP2*, *PtTCP12*, *PtTCP37*, *PtTCP38*, *PtTCP40*, *PtTCP41*, *PtTCP42* and *PtTCP43* were co-expressed with clock genes in diurnal cycle. Only the expression of *PtTCP42* showed diurnal oscillation.

**Conclusions:**

The *TCP* gene family, especially clade II, may play an important role in the regulation of the season and circadian rhythm of *P. tabuliformis*. In addition, the low temperature in winter may affect the diurnal oscillations.

**Supplementary information:**

The online version contains supplementary material available at 10.1186/s12870-022-03554-4.

## Background

Transcription factors (TFs) are proteins that specifically bind to the promoter region of eukaryotic genes. They play important roles in regulating transcriptional initiation of specific sequences that is fundamental to both plant development and responses to the the external environment stimulation [1, 2]. The TCP family is an important type of transcription factors. The domain of the TCP family and its first genes were described in the 1999. The so-called “TCP” was named after the three characterized family members: TEOSINTE BRANCHED1 (TB1) from maize (*Zea mays*), which involved in apical dominance regulation; CYCLOIDEA (CYC) from snapdragon (*Antirrhinum majus*), which controlled floral asymmetry; and the PROLIFERATING CELL FACTORS (PCFs) from rice (*Oryza sativa*), which were essential for meristematic tissue-specific expression [3]. The TCP proteins contain a conserved basic helix-loop-helix (bHLH) motif, known as the TCP domain, which is composed of about 60 amino acids [3]. The TCP domain is important for DNA binding, protein-protein interaction, and subcellular localization [4]. According to the sequences of TCP conserved domain and phylogenetic relationships, the members of the *TCP* gene family always can be divided into two subfamilies: clade I and clade II. Clade II is also known as the PCF subfamily, while clade I *TCP* members are further divided into CIN and CYC/TB1 subfamilies [5, 6]. The most obvious difference between these two subfamilies is that the basic region of TCP domain of clade I subfamily has four amino acids more than that of clade II subfamily. In addition, several members of clade I have another conserved region outside the bHLH domains named the R domain, which is an arginine-rich motif containing eighteen to twenty residues [5]. The R domain may also be involved in protein–protein interactions [7, 8].

The *TCP* gene family has been reported in a number of plant species. For instance, there are 24 *TCP* genes that were found in *Arabidopsis thaliana* [5], 28 in *Oryza sativa* [5], 27 in *Cucumis sativus L.* [9], 30 in *Solanum lycopersicum* [10] and 42 in *Panicum virgatum L.* [11]. The TCP gene family can participate in different processes of plant development, such as seed germination [12], cell proliferation [13, 14], and leaf [15, 16], flower [17], axillary bud [18], lateral branching [19] and pollen development [20]. In addition, the TCP gene family also plays an important role in the response to various abiotic stresses, such as salt stress [11, 21], drought stress [1, 4] and low temperature and short photoperiod [9]. The *TCP* gene family also influence developmental and abiotic stress signaling by hormone pathways [22, 23]. Therefore, these evidences indicate that *TCP* genes play an important role in both plant growth and development and abiotic stress.

In plants, many aspects of their life history were subject to seasonal control, such as germination, leaf growth, flowering and deciduous leaves. In addition, the daily rotation of the earth led to repeated and rhythmic but predictable environmental changes, which led to significant changes in the behavior, physiology and metabolism of most organisms living on the earth between day and night. These diurnal and seasonal changes were considered important traits for their survival and growth and may be under clock control. This endogenous system of organisms that helped predict environmental changes was called the circadian clock [24, 25]. The circadian clock system in plants was often separated into three parts: the input pathway, central oscillator and output pathway. Plants can sense external environmental information such as temperature, light, and nutrition and transfer these signals to central oscillator, which generated a rhythm of the output genes. With the continuous development of biotechnology, researchers have discovered a large number of components functioning in input pathway, central oscillator or output pathway in *Arabidopsis* [26–29]. These circadian clock components interacted to form a complex network. MYB transcription factors CIRCADIAN CLOCK ASSOCIATED 1 (CCA1)/LATE ELONGATED HYPOCOTYL (LHY) and TIMING OF CAB EXPRESSION 1 (TOC1) composed the most characteristic negative feedback loop in the central oscillator, which is critical for its regulation by the clock [30]. *PSEUDO RESPONSE REGULATOR 5/7/9* (*PRR5/7/9*), *EARLY FLOWERING 3* (*ELF3*), *EARLY FLOWERING 4* (*ELF4*), *GIGANTEA* (*GI*), and *LUX ARRHYTHMO* (*LUX*) participated the other interlocked feedback loops in the central oscillator [24, 31]. While *LIGHT-REGULATED WD1* (*LWD1*) and *PRR9* can compose a positive-feedback loop [32]. Stability of *GI* and degradation of *TOC1* were controlled by *ZEITLUPE* (*ZTL*), which may play a role in light input to the clock [33]. In addition, flowering-time genes have been shown to be widely involved in *Arabidopsis* clock systems, such as *ELF3* and *GI*. Transcript levels of *CONSTANS-LIKE 1* (*COL1*) and *CONSTANS-LIKE 2* (*COL2*) in *Arabidopsis* were also under circadian clock control, and over-expression of *COL1* affected two distinct circadian rhythms [33].

In *Arabidopsis*, AtTCP21, which known as CHE (CCA1 HIKING EXPEDITION), interacted with TOC1 and bound to the CCA1 promoter to repress its gene expression [34]. AtTCP2, AtTCP3, AtTCP11 and AtTCP15 were found to interact with different components of the core circadian clock, and *tcp11* and *tcp15* mutants showed altered transcript profiles for several core clock components [35, 36]. AtTCP20 and AtTCP22 also could direct interaction with LWD1, then associate with the CCA1 promoter in vivo and promote the expression of *CCA1*, which could sustain a robust clock [37]. However, the relationship between the TCP family and periodic growth has not been systematically studied, especially in the conifers that don’t fall in winter.

Japanese cedar has a distinct circadian rhythm in summer, in which stress-related signaling pathways (such as ABA-related genes) showed particularly strong rhythmic oscillations [38]. The clock genes adapted to the harsh daytime environment in summer by regulating the transcription of stress-related genes. In Douglas-fir, 58.7% of the expressed transcripts exhibited significant annual cycles and 29% exhibited significant diurnal cycles, with thousands of genes reaching their annual peaks of activity during winter dormancy [39]. The regular oscillation of plant genes makes it end the growth period in time and enter dormancy. Photosynthesis is carried out under favorable conditions, providing protection during the dormant period and effectively avoiding the occurrence of problems such as frost damage.

*P. tabuliformis* is an important economic tree species in northern China, which is an evolutionarily old conifer genus. Genomewide analysis of the presence of TCP transcription factors in *P. tabuliformis* would be necessary for *P. tabuliformis* growth rhythm research. In this study, a total of 43 *TCP* members were identified in the *P. tabuliformis* genome. We analyzed the phylogenetic relationships, multiple comparison sequences, gene structure, conserved motifs, domain and chromosomal location distribution. We built co-expression networks in annual cycle and diurnal cycle. The effects of *TCP* genes on seasonal and diurnal growth rhythm were analyzed to enhance our understanding of molecular mechanisms of diurnal and seasonal adaptation in conifers.

## Results

### Identification of *TCP* genes in *P. tabuliformis*

Based on the *P. tabuliformis* genome database [40], a total of 43 putative TCP transcription factors were identified (Table S 1). With the online program SMART and NCBi CDD, we identified all proteins that contained a conserved TCP domain (Fig. 3C and Table S 2), which were named as PtTCP1 to PtTCP43. Biochemical properties of PtTCP members were globally analyzed (Table S 3). The lengths of these predicted PtTCP peptides ranged from 122 (PtTCP13) to 723 (PtTCP9) amino acids and molecular weight from 13159.82 (PtTCP13) to 80846.73 (PtTCP9) Da. The isoelectric point (PI) varied from 5.39 (PtTCP15 and PtTCP16) to 9.92 (PtTCP10). The value of the aliphatic index ranged from 52.65 to 97.54, which suggested that these predicted PtTCP proteins contained rich aliphatic amino acids. The GRAVY of all PtTCP proteins was negative value, indicating that PtTCPs were hydrophilic.

**Fig. 1:**
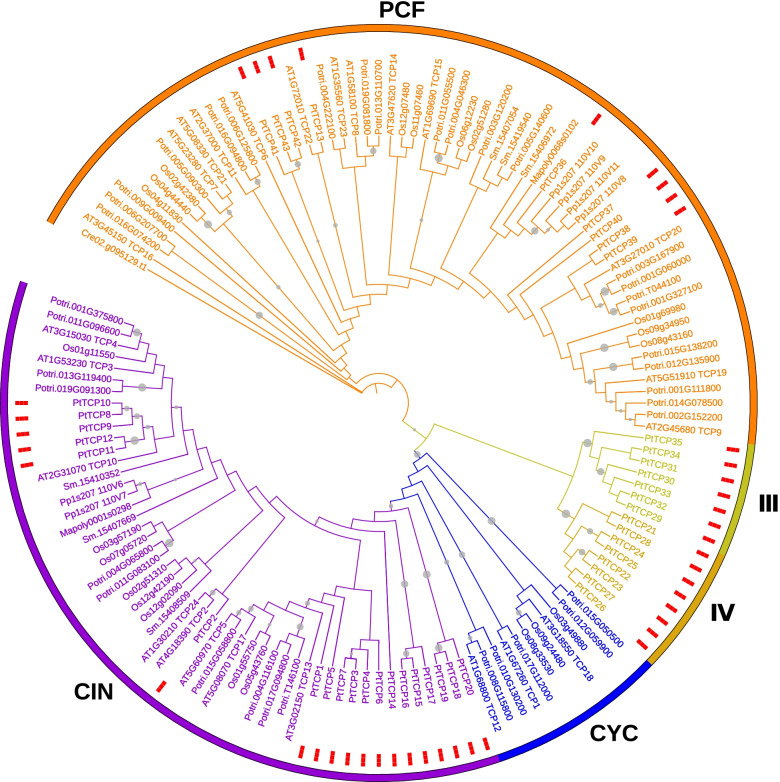
Phylogenetic analysis of TCP genes between *P. tabuliformis*, *Chlamydomonas reinhardtii*, *Marchantia polymorpha*, *Selaginella moellendorffii*, *Physcomitrella patens*, *Oryza sativa*, *Populus trichocarpa* and *Arabidopsis*. Different subfamilies were indicated in a specific colour. Genes of *P. tabuliformis* were marked with small red squares

### Phylogenetic analysis of TCP genes among plants

To further understand the evolutionary relationship of the *TCP* genes in plants and classify the candidate *TCP* genes in *P. tabuliformis*, we constructed a comprehensive phylogenetic tree with maximum likelihood methods of the eight representative species, including *Chlamydomonas reinhardtii* (green algae), *Marchantia polymorpha* (liverwort), *Selaginella moellendorffii* (selaginella), *Physcomitrella patens* (moss), *Oryza sativa* (rice), *Populus trichocarpa* (polar), *Arabidopsis thaliana* and *P. tabuliformis* (Fig. 1, Table S4.). At the same time, we have added six species to increase the accuracy of the phylogenetic tree, including *Amborella trichopoda*, *Sorghum bicolor (L.) Moench*, *Ginkgo biloba L.*, *Picea abies (L.) Karst.*, *Pinus taeda L.* and *Pinus lambertiana Douglas* (Fig S1). The relationship of TCPs in *P. tabuliformis* was consistent with the previous results, which proves that our classification results are reliable. In addition, we also provide a species tree to illustrate the relationship of the selected species (Fig S2).

**Fig. 2:**
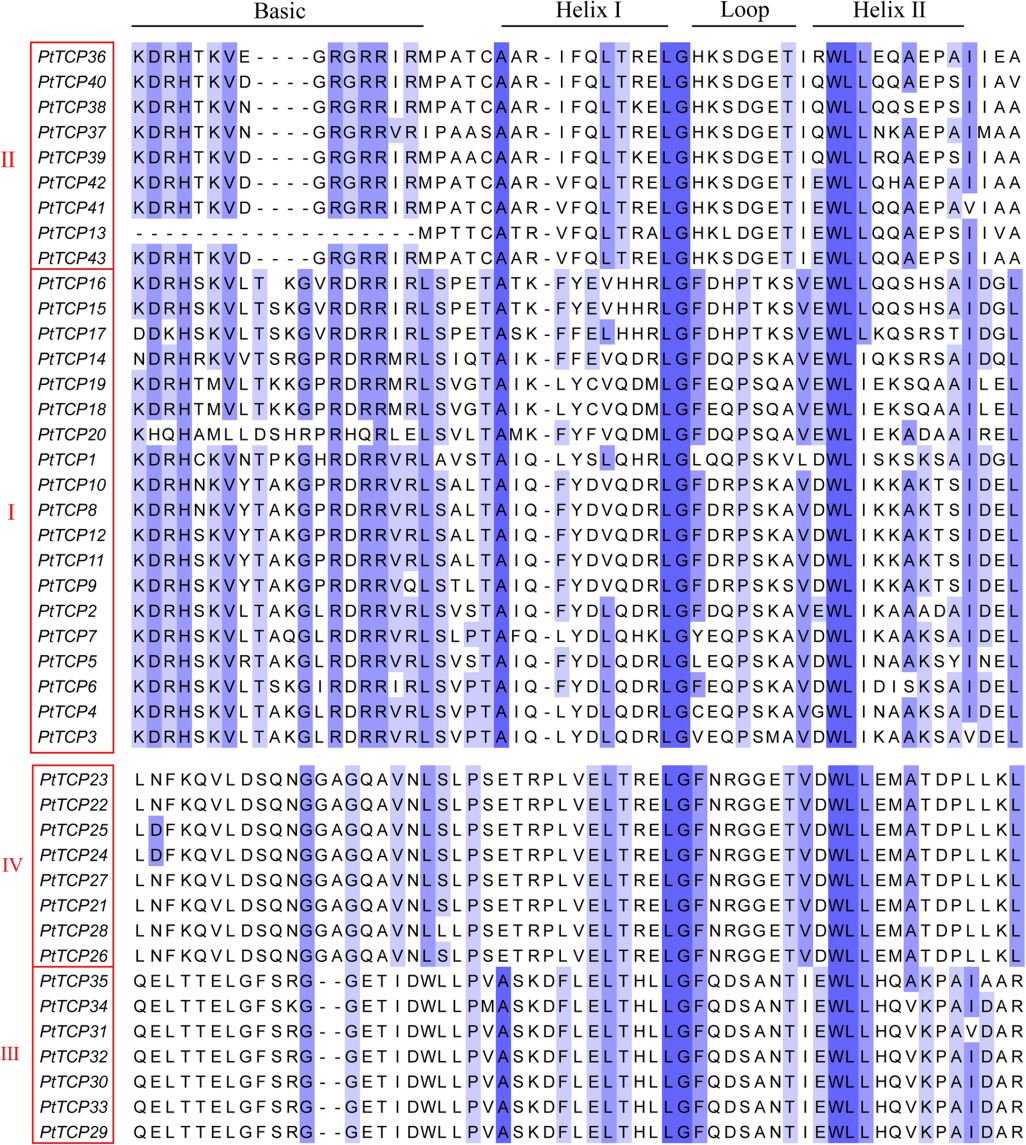
Multiple alignments of TCP family members in *P. tabuliformis*. Overall conserved amino acids were shaded in blue. The Basic, Helix I, Loop and Helix II regions were indicated

In the phylogenetic tree, the TCP proteins of all the eight species were classified into four classes. All the AtTCPs of Arabidopsis and OsTCPs of rice in this phylogenetic tree belonged to the same classification as previous studies [5, 41], supporting its reliability. Nineteen *PtTCPs* were classified into clade I, accounting for almost half of the entire family .The clade I group was further divided into two subfamilies: clade CIN and clade CYC/TB1. Nine *PtTCPs* were classified into clade II, which was named PCF. Seven *PtTCPs* were classified into clade III and the rest eight *PtTCPs* were classified into clade IV. The *AtTCP* family members were mostly concentrated in clade PCF, clade CIN members were few, and clade CYC members were the least in Arabidopsis. However, the *PtTCP* members were mainly concentrated in clade CIN. There was no member distribution in clade CYC, and relatively few members in clade PCF.

The TCP proteins generally present several typically conserved domain features, basic, helix1, loop, and helix2 domains, which form a special bHLH structure with approximately 60 amino acid residues [3]. The sequence alignment analysis shows that almost clade I and II PtTCP proteins contain the conserved bHLH domain, but the members that belonged to clade II (PCF) have four amino acid deletions in the bHLH domain compared with clade I (CYC/TB1 and CIN) (Fig. 2). This result was consistent with the phylogenetic analysis. Clade III and IV PtTCP proteins don’t contain the conserved bHLH domain. Clade III and clade IV are specific subfamilies of *P. tabuliformis*, which angiosperms may have lost during long-term evolution. Members that belonged to clade III have a two amino acid deletion compared with clade IV, which was also consistent with the results of our phylogenetic analysis.

**Fig. 3:**
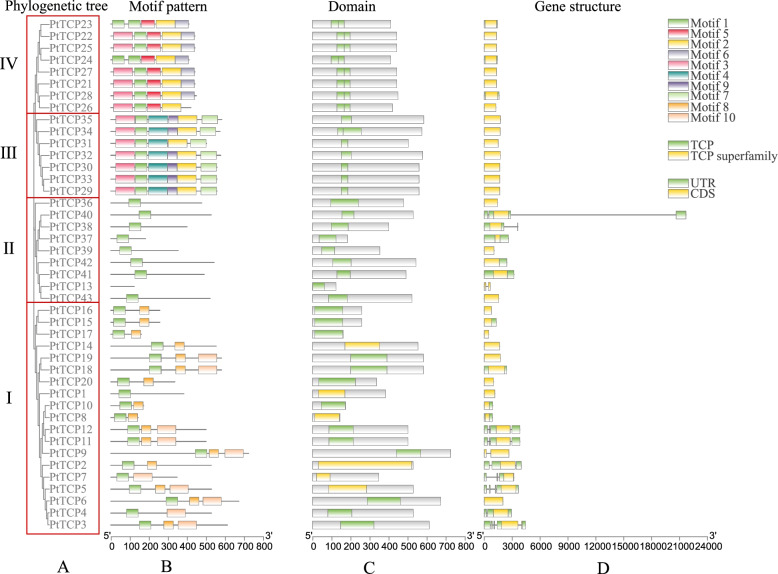
Phylogenetic relationships, domain, gene structure, and architecture of conserved protein motifs in PtTCP family. A. Estimated phylogeny of PtTCP genes. B. Different motifs were represented by different colors. The black lines represented the non-conserved sequences. Lengths of motifs for each PtTCP protein were displayed proportionally. C. TCP domains were represented by green, TCP superfamily were represented by yellow. D. UTR were represented by green, CDS were represented by yellow. Nucleic acid lengths are indicated by the scale at the bottom

### Gene structures, domain and conserved motifs characterization of PtTCPs

We used the conserved TCP domain sequences of PtTCP proteins to construct a new phylogenetic tree (Fig. 3A), and the result showed that it was also divided into four subclades. Generally, conserved functionally motifs in same TF families are likely to share similar functions within a group in a phylogenetic tree. To further investigate the characteristic regions of 43 PtTCP proteins, the conserved motifs were analyzed and ten motifs were identified in PtTCPs using the MEME tool (Fig. 3B). Except for PtTCP13, almost all PtTCPs contained motif 1, which indicates that this motif has the basic TCP domain with a typical function. Except for PtTCP1, PtTCP 4 and PtTCP 7, clade I proteins contained motif 8 and several clade I genes also contained motif 10 (PtTCP3-7, PtTCP9, PtTCP11-12, PtTCP18-19). But clade III and IV proteins contained more other motifs. They both contained motif 2 and motif 3 expect for PtTCP23 and PtTCP24. In addition, clade III proteins also contained motif 4, motif 7 and motif 9 expect for PtTCP31. Clade IV proteins also contained motif 5 and motif 6 expect for PtTCP26.As expected, the results from the conserved motif analysis clearly distinguished four subfamilies. Similar motif compositions were identified in most close relatives from subfamilies, suggesting that PtTCPs in same subfamily may perform the similar function and that some of motifs may play a vital role in specific function.

To characterize the structural diversity of *PtTCP* genes, the distribution of gene structures of each *PtTCP* gene was further analyzed (Fig. 3D). Through gene structure analysis, differences between the four subfamilies could be observed, and there were also differences in the number of introns between genes in different subfamilies. Clade I had eight genes with introns, accounting for about half of clade I. One gene contained four introns, three contained three introns, two contained two introns and two contained one intron. Clade II had only three genes (*PtTCP13*, *PtTCP38*, *PtTCP40*) with introns. One gene contained two introns and two contained one intron. But clade III and clade IV had no introns. Most *PtTCP* genes in the same subfamily shows similar exon/intron distribution patterns, which supported the classification relationship of subclades.

### Chromosome distribution of TCP genes in *P. tabuliformis*

**Fig. 4:**
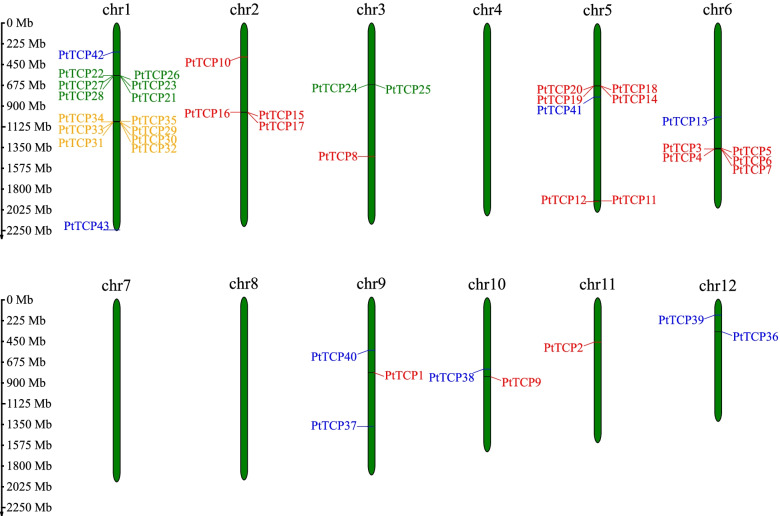
Schematic representations for the chromosomal distribution of *TCP* genes in *P. tabuliformis*. A total of 43 TCP family genes were anchored onto 12 chromosomes of *P. tabuliformis*. The scale on the left represented the physical length of the chromosomes; Mb = million base pair. The clade I genes were shown in red, clade II in blue, clade III in yellow, and clade IV in green

The 43 identified *PtTCP* genes were mapped to 12 chromosomes (Fig. 4). The distribution of Clade I and Clade II genes on chromosomes were relatively scattered. Clade I genes had different distribution on seven chromosomes, while Clade II genes had different distribution on six chromosomes. All Clade III and most clade IV genes (except for *PtTCP24* and *PtTCP25*) were distributed on Chr1. The duplicated genes which can be classified into five different categories, namely, whole-genome duplicates (WGD), tandem duplicates (TD), proximal duplicates (PD), transposed duplicates (TRD), and dispersed duplicates (DSD) [42]. Our result showed that only the Clade III genes from TD, while the clade IV paralogs originated primarily from DSD.

#### Co-expression networks in annual cycle and diurnal cycle

**Fig. 5:**
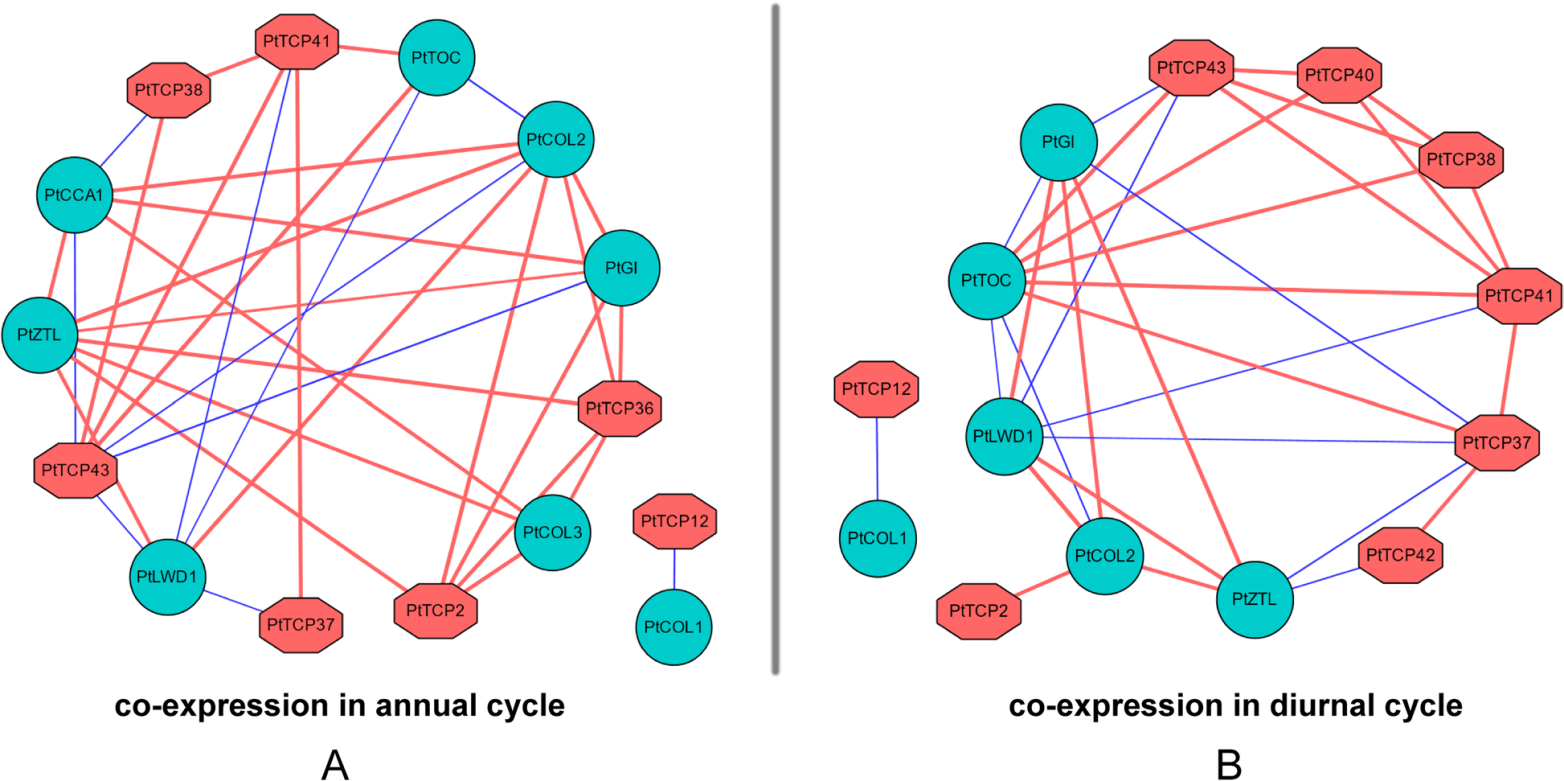
Estimated gene network including *PtTCPs* and core clock genes in annual cycle (**A**) and diurnal cycle (**B**). The clock genes of *P. tabuliformis* were in the blue box, the TCP family genes were in the red box. The red lines indicate a positive relationship between two genes, and the blue lines indicate a negative relationship between two genes

Homologs of clock genes have been identified and they are very conserved in conifer [38]. In the *P. tabuliformis* genome project, the photoperiodic pathway is the one of the most conserved pathways during the seed plant evolution [40]. In present study, we monitored *PtCCA1*, *PtTOC*, *PtLWD1*, *PtGI*, *PtZTL*, *PtCOL2* and *PtCOL3* from *P. tabuliformis* and we monitored their diurnal cycle expression profiles and annual cycle expression profiles twice a month for two years by RNA-seq (Table S5, Fig S3, Fig S4). They all showed obvious seasonal oscillations and some genes showed diurnal oscillations, which further confirmed the conservation of clock genes in conifer. To analyze the relationship between the *TCP* family of *P. tabuliformis* and circadian clock genes, we built co-expression networks (Fig. 5). Six *PtTCPs* (*PtTCP2*, *-36*, *-37*, *-38*, *-41*, *-43*) were identified as co-expressing with seven putative clock component genes, including *PtCCA1*、*PtTOC*、*PtLWD1*、*PtGI*、*PtZTL*、*PtCOL2* and *PtCOL3* in annual cycle (Fig. 5A). In the estimated network, the expression of most genes were positively correlated, and a few genes were negatively correlated. Interestingly, *PtTCP12* and *PtCOL1* were co-expressed independently. Similarly, seven *PtTCPs* (*PtTCP2*, *-37*, *-38*, *-40*, *-41*, *-42*, *-43*) were identified as co-expressing withfive putative clock component genes, including *PtTOC*、*PtLWD1*、*PtGI*、*PtZTL* and *PtCOL2* in diurnal cycle (Fig. 5B). In addition, *PtTCP12* and *PtCOL1* were also co-expressed independently. The results of co-expression of the TCP gene family and clock genes indicated that *PtTCPs* may function as circadian clock genes and play an important role in the growth and development of *P. tabuliformis.*

### The expression of several *PtTCPs* showed season oscillations

**Fig. 6:**
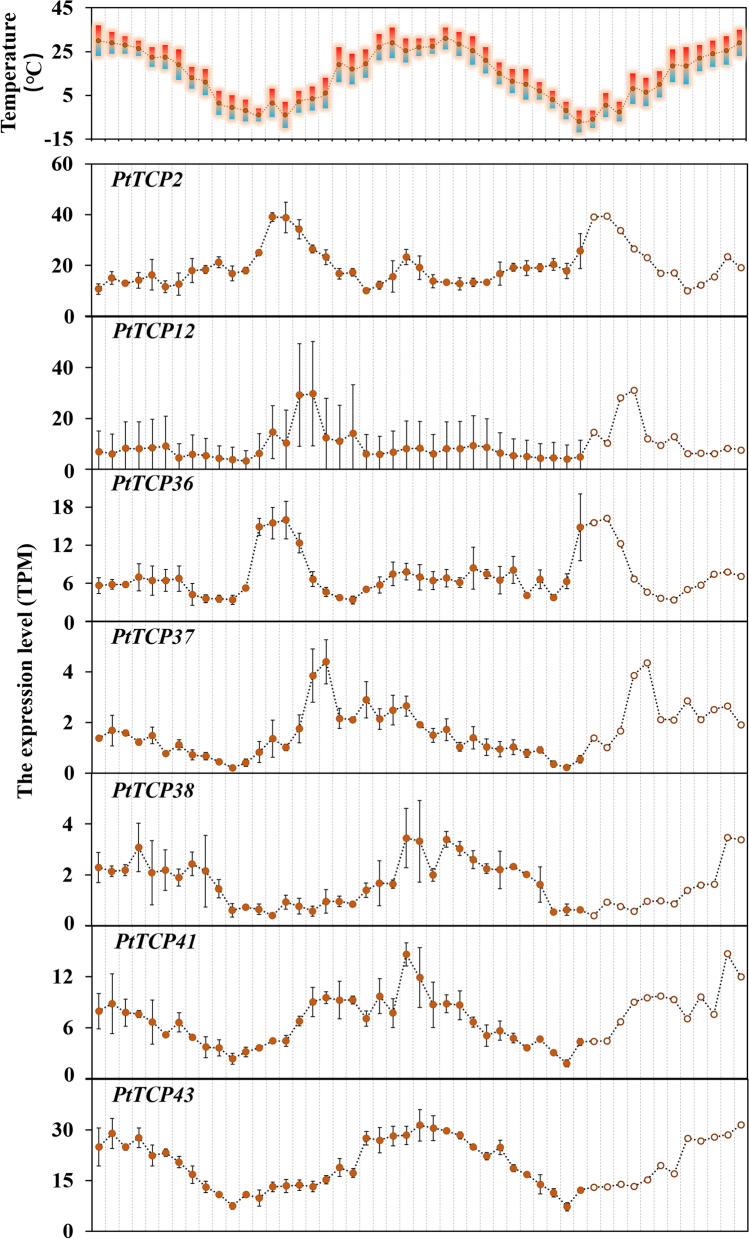
The expression of *PtTCP2*, *PtTCP12*, *PtTCP36*, *PtTCP37*, *PtTCP38*, *PtTCP41* and *PtTCP43*. Monitoring lasts for two years, from July 1, 2017 to July 1, 2019. Error bars represented variability of three independent replicates

To further explore the relationship between the *TCP* gene family and the circadian clock, we monitored their annual cycle expression profiles twice a month for two years by RNA-seq (Fig. 6). We found that the expression of *PtTCP2*, *PtTCP12*, *PtTCP36*, *PtTCP37*, *PtTCP38*, *PtTCP41* and *PtTCP43* both showed obvious seasonal oscillations. The expression of *PtTCP2* and *PtTCP36* peaks in February each year, *PtTCP12* and *PtTCP37* in March, *PtTCP38* and *PtTCP41* in June, and *PtTCP43* in July. This further verified that the PtTCP family plays an important role in the regulation of the circadian clock of *P. tabuliformis.*

### The expression of *PtTCP42* showed diurnal oscillations

**Fig. 7:**
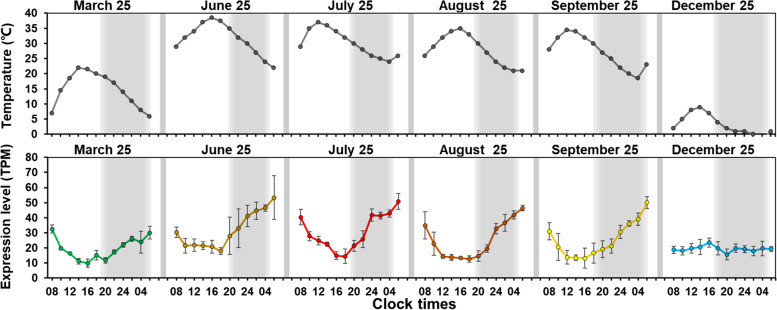
The expression of *PtTCP42* at 8:00, 12:00, 16:00, 20:00, 24:00 and 4:00 in March 25, June 25, July 25, August 25, September 25 and December 25. Error bars represented variability of three independent replicates

Data revealed significant oscillations in the expression of *PtTCP42* in March 25, June 25, July 25, August 25 and September 25, except for December 25. The level of transcription in March 25 reached a peak at 8:00 and subsequently declined (Fig. 7). The level in June 25, July 25, August 25 and September 25 both reached a peak at 4:00. The level in March 25 and September 25 reached a bottom at 16:00. The level in June 25 and July 25 reached a bottom at 18:00. The level in August 25 remained at the minimum value from 12:00 to 20:00. The expression of PtTCP42 in March 25, June 25, July 25, August 25 and September 25 both showed diurnal expression patterns. But in December 25, the expression of *PtTCP42* only slightly decreased at 20:00, indicating that low temperature may affect its diurnal rhythm. But other genes didn’t show obvious diurnal oscillations.

## Discussion

The *TCP* gene family is a type of plant-specific transcription factors. To date, many *TCP* genes have been reported in a wide range of plant species, such as *Arabidopsis* [43], legume [1], tomato [10], *Panicum virgatum L.* [11], *ziziphus jujuba* [2], grapevine [4], *solanum tuberosum* [44]. However, no systematic and comprehensive information of the *TCP* gene family in *P. tabuliformis* have been done. In the present study, we performed a comprehensive analysis of the PtTCP family in *P. tabuliformis* by analyzing their isoelectric point (pI), molecular weight (MW), phylogenetic relationships, multiple comparison sequences, gene structure, conserved motifs, domain and chromosomal location distribution. The systematic characterization of *PtTCP* genes in *P. tabuliformis* will provide a better foundation for further functional studies of this gene family during *P. tabuliformis* growth and development.

In most species such as *Arabidopsis* and rice [5, 41], the TCP gene family is generally divided into two classes. The clade I group was further divided into two subfamilies: clade CIN and clade CYC/TB1. The clade II was also known as clade PCF. But in our study, phylogenetic analysis (Fig. 1) and sequence alignment (Fig. 2) showed the TCP genes in *P. tabuliformis* were divided into four classes. In addition to clade I and clade II, it also added clade III and clade IV. The origin of clade CYC/TB1 members has occurred later than clade CIN members in angiosperms [11], which explains that the PtTCP genes in clade I were concentrated in clade CIN, while clade CYC had no member distribution. However, clade III and clade IV didn’t appear in most angiosperm such as Arabidopsis. Gymnosperms and angiosperms separated 300 million years ago, probably because angiosperms lost them over a long period of evolution. In general, transcription factor families keep evolving in response to environmental changes, with proteins transforming from simple to complex. The number of motifs in a protein reflects the evolution of function. PtTCP members of the same group have similar motif distributions (Fig. 3B), so they may have similar functions. In summary, multiple comparison results, motif location and gene structures of PtTCP members were roughly conserved in the same clade but showed significant distinction among different clades, which further support the reliability of our phylogenetic analysis.

In the present study, a total of 43 *PtTCP* genes were identified from the *P. tabuliformis* genome. *P. tabuliformis* contained approximately twice as many TCP proteins as Arabidopsis and rice, which had 24 and 21 TCP members, respectively, implying that *TCP* genes in various plants have expanded to different degrees. Combined with genome size, it was found that the number of *TCP* family members was not related to the genome size. For example, the genome size of tomato is 960 Mb with only 30 *TCP* members, while the genome size of apple is 742 Mb with 52 *TCP* members [45, 46]. The diversity of the number of *TCP* family members in different species may be influenced by genome duplication events, such as whole genome duplication, segmental duplication or tandem duplication [7]. We intended to seek evolutionary relics of WGD in *P. tabuliformis* by detecting paralogous synteny gene blocks among different chromosomes. However, we only identified 65 blocks and 857 syntenic gene pairs based on all-to-all blastp alignments (The number of syntenic gene pairs in other conifers is less than this, so that it is impossible to carry out subsequent comparative analysis), there does not include the TPC family genes. These pieces of evidence indicate that the paleopolyploidy was occurred in very ancient time and only some remnants can be identified (only account for 0.6% in all genes in the genome) [40]. So, the genome duplication pairs and synteny analysis cannot be done.

Previous studies have shown that the *TCP* gene family plays an important role in the growth and development of plants [13, 16, 17]. And studies also have reported that the *TCP* family plays an important role in the clock system in *Arabidopsis*. But there was no report of circadian clock genes in *P. tabuliformis*. In *Arabidopsis*, AtTCP11, -15, -20, -21, -22 of clade II subfamily interacted with multiple clock genes and may played a role in the developmental regulation [34–37]. When we analyzed the TCP family of *P. tabuliformis*, we found that the clade II subfamily (*PtTCP36*, *-37*, *-38*, *-41*, *-43*) was co-expressed with clock genes (Fig. 5), and its annual monitoring results also showed seasonal oscillations (Fig. 6), which proved that the function of the TCP clade II family are relatively conserved. As perennial species, periodic growth is essential for tree survival and growth. In general, increased plant tolerance to abiotic stress is associated with increased nutrient uptake, altered hormonal balance, enhanced reactive oxygen species scavenging system activity, and osmotic regulator synthesis, while the circadian clock controls plant nutrient homeostasis, hormone synthesis and signaling, redox reactions, and changes in the concentration of some major osmo-regulatory substances [47–51], which suggests that plants circadian system plays an important role in the face of abiotic adversity. We reported the expression patterns of all TCPs at the genome-wide level of a conifer species for the first time, providing overview data for subsequent studies of gene functions in conifers. Overexpression and customizable genome-editing analysis of these genes in conifer could be helpful to fully understanding the underlying molecular mechanisms about this issue. Through the in-depth study of the TCP family, the ability to artificially regulate the circadian rhythm mechanism in plants is expected to make crops more productive and more resistant to harsh environments, so that they can thrive in a variety of external environments.

Interestingly, *PtTCP42* showed obvious circadian rhythm. But in December 25, the expression of *PtTCP42* only slightly decreased at 20:00 (Fig. 7). This indicated that the low temperature in winter may have affected the circadian rhythm of *PtTCP42*. Studies have found that the seasonal changes of plants affected the clock components and thus affected the circadian rhythm [52, 53]. In rice, core clock component genes *OsLHY* and *OsPRR1* were regulated by chilling stress [54]. Circadian clock behavior was disrupted by cold temperatures and the primary oscillator feedback loop was not functional at 4 °C in the chestnut tree (*Castanea sativa*) [55, 56]. Dampening of diurnal rhythms in winter indicated the rhythm can change seasonally with environmental conditions in Japanese cedar (*Cryptomeria japonica (L.f.) D.Don*) [38]. The expression of clock genes may be influenced by seasonal environmental changes and consequently lead to activation of downstream pathways that contribute to freezing tolerance, which is important for survival of tree species in winter. In addition, clock genes, in turn, can affect the freezing resistance of plants. In *Arabidopsis*, core clock components *CCA1* and *LHY* regulated expression of the CBF (C-REPEAT BINDING FACTOR) pathway, which has a major role in cold acclimation [57]. Reducing the expression of *PttLHY* genes compromised freezing tolerance in Populus trees [58]. Although conifers are evergreen species, their periodic growth traits, such as cold domestication, are important breeding goals and ecological conservation goals [59]. *P. tabuliformis* has strong adaptability and is extremely resistant to low temperature. However, the molecular mechanism of cold resistance of *P. tabuliformis* has not been analyzed so far. The study of the TCP family is helpful to analyze the cold resistance mechanism of *P. tabuliformis*, and provides theoretical support for further grasping its growth physiology, ecological characteristics and expanding its introduction.

## Conclusions

Our results provide a foundation for future functional studies to determine the molecular mechanisms of *TCP* genes in the development of *P. tabuliformis*. In this study, 43 *PtTCP* genes were identified from the *P. tabuliformis* genome, which were distributed on 12 chromosomes. Based on the phylogenetic tree, all the *TCP* genes were divided into four subfamilies. The *TCP* genes from the same evolutionary branches shared similar motifs. Most genes had no introns. Co-expression network indicated that *PtTCP2*, *PtTCP12*, *PtTCP36*, *PtTCP37*, *PtTCP38*, *PtTCP41* and *PtTCP43* were co-expressed with clock genes in annual cycle and their annual cycle expression profiles both showed obvious seasonal oscillations. *PtTCP2*, *PtTCP37*, *PtTCP38*, *PtTCP40*, *PtTCP41*, *PtTCP42* and *PtTCP43* were co-expressed with clock genes in diurnal cycle. Only the expression of *PtTCP42* showed diurnal oscillation and the low temperature in winter may have affected its diurnal rhythm. The study of *PtTCP* gene family was helpful to the understanding of the relationship between circadian clock and cold resistance, but how *PtTCPs* connect with clock component genes and play a role still needs further research.

### Methods

#### Plant materials and sample collection

Seasonal samples of *Pinus tabuliformis* were collected by J-J.M. from individual trees at the botanical gardens of Beijing Forestry University in Beijing, China (116°33.91160E, 40°00.08610 N and 44 m above sea level). The other plant materials of *P. tabuliformis* were obtained by S-H.N. from a seed orchard which belong to a Chinese pine breeding program located in Pingquan City, Hebei Province, China (118°44.6758′ E, 40°98.8784′ N, 560 − 580 m above sea level) (no any required permission for its sample collection and use). The transcriptional expression of *P. tabuliformis* was dynamically monitored for two years. From July 2017 to July 2019, current year needles were collected twice a month about every two weeks. A total of 147 samples (49 time points × three biological replicates) for annual cycle expression analysis were collected around 12 o ‘clock in the afternoon on clear days. The needles of *P. tabuliformis* were collected at 8, 12, 16, 20, 24 and 4 o’clock on March 25, June 25, July 25, August 25, September 25 and December 25, 2020, respectively. Then, the collected needles were quickly placed in liquid nitrogen and stored at -80°C for total RNA extraction. Three different trees were used as biological replicates for RNA-seq analysis.

 All experimental research and field studies on plants (either cultivated or wild), including the collection of plant material, comply with relevant institutional, national, and international guidelines and legislation, as established by the State Forestry and Grassland Administration of China.

### Identification of *TCP* family members in *P. tabuliformis*

The protein sequences of TCP family in *Arabidopsis* were downloaded from the iTAK database (http://itak.feilab.net/cgi-bin/itak/index.cgi).These sequences were used to search from our in house *P. tabuliformis* reference genome database using the local blast program and the E-value cut-off was set as 1e-6. The conserved TCP domains of PtTCPs were further confirmed using the NCBI CDD tool (https://www.ncbi.nlm.nih.gov/Structure/bwrpsb/bwrpsb.cgi). SMART was used to confirm the domains(http://smart.embl-heidelberg.de/). Finally, 43 putative *TCP* genes were identified. Meanwhile, information about each *PtTCP* gene, including the protein length, molecular weight (MW), isoelectric point (pI) was acquired from the ExPASy (http://www.expasy.org/tools/).

### Phylogenetic analysis and multiple alignments

The TCP protein sequences of *Chlamydomonas reinhardtii* (green algae), *Marchantia polymorpha* (liverwort), *Selaginella moellendorffii* (selaginella), *Physcomitrella patens *(moss), *Oryza sativa* (rice), *Populus trichocarpa* (polar), *Amborella trichopoda*, *Sorghum bicolor (L.) Moench*, *Ginkgo biloba L.*, *Picea abies (L.) Karst.*, *Pinus taeda L.*, *Pinus lambertiana Douglas* and *Arabidopsis* downloaded from the iTAK database were used for phylogenetic analysis. Multiple sequence alignments of *P. tabuliformis* and other plants TCP proteins were performed using MUSCLE with default parameters. A phylogenetic tree was subsequently constructed using the Maximum Likelihood method of MEGA7 with 200 bootstrap replications. The phylogenetic tree constructed by MEGA was uploaded to iTOL (http://itol.embl.de/) for further editing. Multiple sequence alignments of the identified *P. tabuliformis* TCPs were constructed using ClustalX (http://www.clustal.org/clustal2/). The species tree was generated at http://timetree.org/.

### Gene structure, domain and conserved motifs characterization

Gene structure was investigated using TBtools software [60]. Conserved domain identification was performed using NCBI CCD online search (https://www.ncbi.nlm.nih.gov/Structure/bwrpsb/bwrpsb.cgi). SMART was used to confirm the results (http://smart.embl-heidelberg.de/). Motif detection was predicted using the online tool MEME Version 5.3.2 program (https://meme-suite.org/meme/tools/meme). The TBtools software was used to integrate phylogenetic trees, conserved motifs, domains and gene structure results.

### Chromosome distribution

The chromosomal distribution information of the identified *TCP* genes was searched from the *P. tabuliformis* genome database using the TBtools software, and the results obtained were visualized using MG2C v 2.1 online tools (http://mg2c.iask.in/mg2c_v2.1/). Based on the previously published method, the duplicated genes which can be classified into five different categories, namely, whole-genome duplicates (WGD), tandem duplicates (TD), proximal duplicates (PD), transposed duplicates (TRD), and dispersed duplicates (DSD) [42]. We defined tandem duplicated pairs as a genomic region harboring three or more neighboring genes, and those genes form a “cluster” on the chromosome.

### Transcriptome data source and expression analysis of *PtTCP* genes

Total RNA from different samples of *P. tabuliformis* were extracted by the Trizol method (Invitrogen, CA, USA). The cleaved RNA fragments were then reverse-transcribed to create the final complementary DNA (cDNA) libraries using the mRNA-Seq sample preparation kit (Illumina, Inc., San Diego, CA, USA). The cDNA libraries were sequenced on the Illumina NovaSeq platform (2 × 150 bp) by using the paired-end module. Clean reads for each sample were aligned to the *P. tabuliformis* reference transcriptome [61].

### Network construction

The similarity distance is characterized by the Pearson correlation coefficient (Pcc) [62, 63], and looped using Bioperl software Iterative calculation [64], set the Pcc domain value to -0.92/0.92 (it is generally considered that the absolute value > 0.8 is a strong correlation between samples). The intergene correlation coefficient matrix was visualized using Cytoscape software [65].

## Supplementary Information

**Additional file 1.**

**Additional file 2.**

**Additional file 3.**

**Additional file 4.**

**Additional file 5.**

**Additional file 6.**

**Additional file 7.**

**Additional file 8.**

**Additional file 9.**

## Data Availability

Any additional information required to reanalyze the data reported in this work paper is available from the Corresponding Author upon request.
