# Supplementary material for: Genome-wide TCP transcription factors analysis provides insight into their new functions in seasonal and diurnal growth rhythm in Pinus tabuliformis
Source: BMC Plant Biol. 2022 Apr 2;22:167. doi: 10.1186/s12870-022-03554-4 (PMC8976390; doi:10.1186/s12870-022-03554-4)
Supplement: Supplementary file 5 — Additional file 5. [file 12870_2022_3554_MOESM5_ESM.docx]

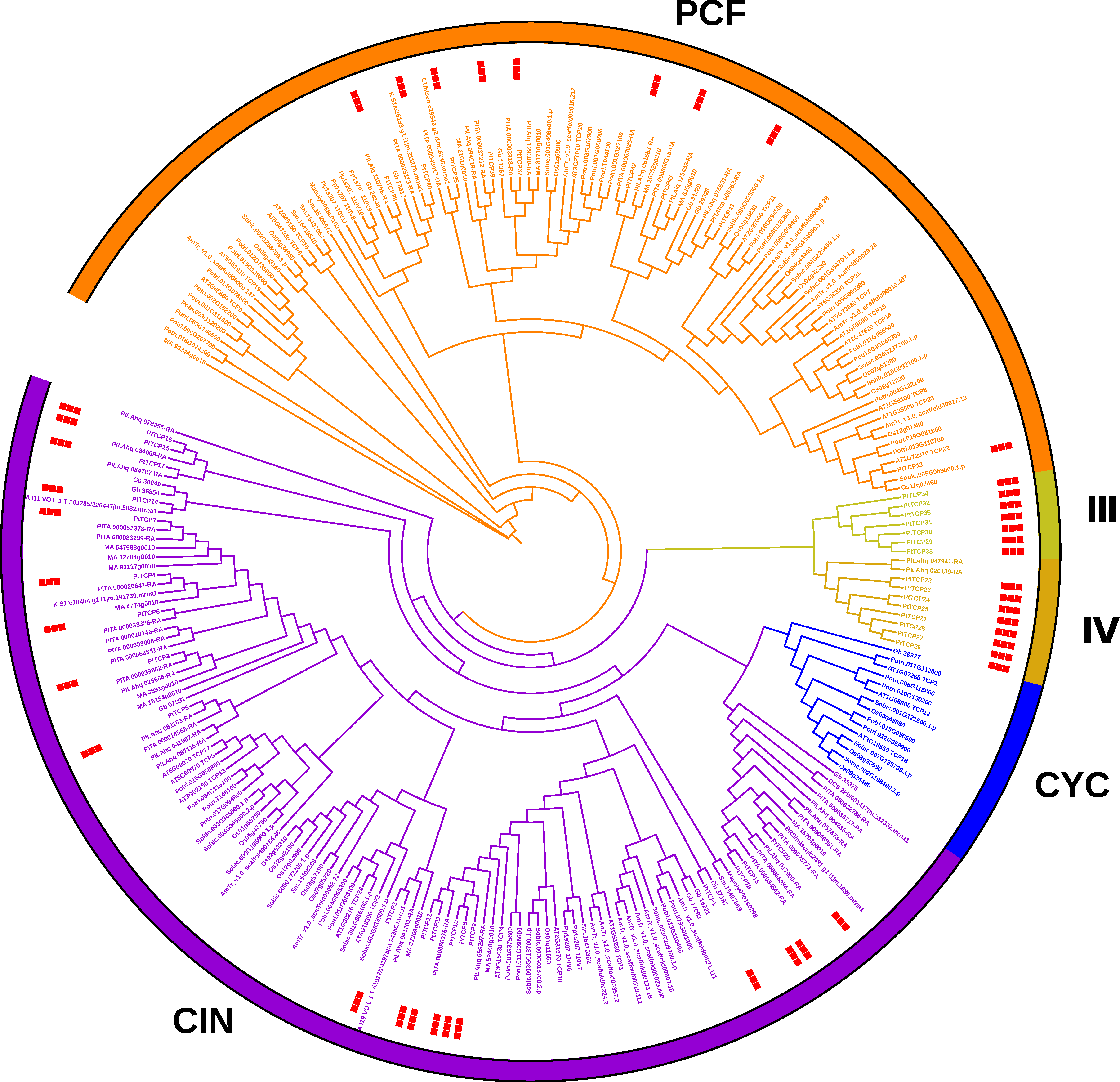


Fig S1. Phylogenetic analysis of *TCP* genes between *P. tabuliformis*, *Chlamydomonas reinhardtii*, *Marchantia polymorpha*, [*Selaginella moellendorffii*](http://itak.feilab.net/cgi-bin/itak/db_family.cgi?plant=88036), *Physcomitrella patens*, *Oryza sativa*, *Populus trichocarpa*, *Amborella trichopoda*, *Sorghum bicolor (L.) Moench*, *Ginkgo biloba L.*, *Picea abies (L.) Karst.*, *Pinus taeda L.*, *Pinus lambertiana Douglas* and *Arabidopsis*. Different subfamilies were indicated in a specific colour. Genes of *P. tabuliformis* were marked with small red squares.
