# Supplementary material for: Genome-wide TCP transcription factors analysis provides insight into their new functions in seasonal and diurnal growth rhythm in Pinus tabuliformis
Source: BMC Plant Biol. 2022 Apr 2;22:167. doi: 10.1186/s12870-022-03554-4 (PMC8976390; doi:10.1186/s12870-022-03554-4)
Supplement: Supplementary file 6 — Additional file 6. [file 12870_2022_3554_MOESM6_ESM.docx]

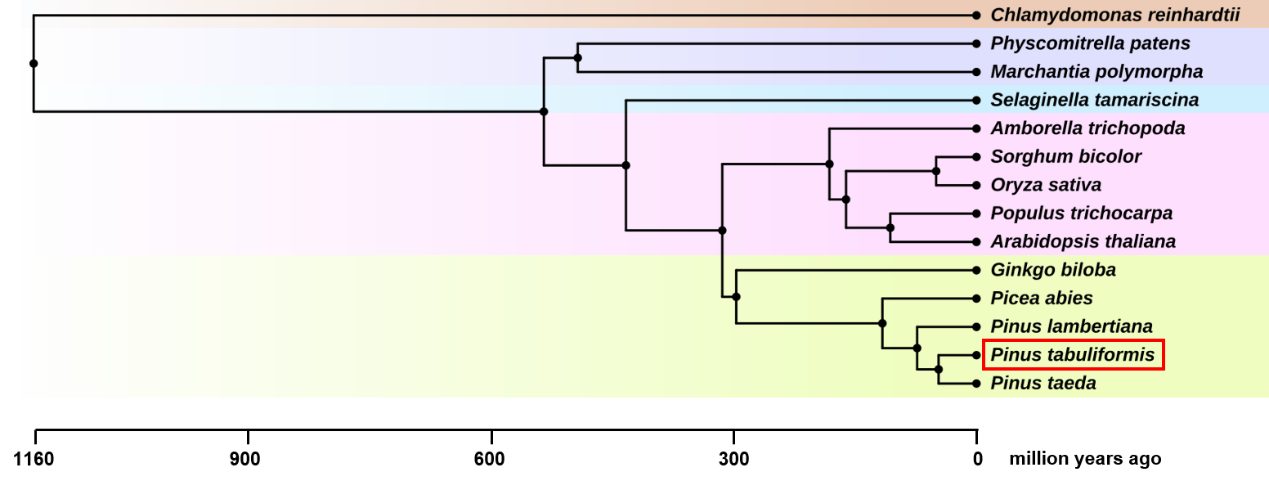


Additional file 6: Fig S2. The evolutionary relationship of 14 species, including *P. tabuliformis*, *Chlamydomonas reinhardtii*, *Marchantia polymorpha*, [*Selaginella moellendorffii*](http://itak.feilab.net/cgi-bin/itak/db_family.cgi?plant=88036), *Physcomitrella patens*, *Oryza sativa*, *Populus trichocarpa* , *Amborella trichopoda*, *Sorghum bicolor (L.) Moench*, *Ginkgo biloba L.*, *Picea abies (L.) Karst.*, *Pinus taeda L.*, *Pinus lambertiana Douglas* and *Arabidopsis.*
