# Supplementary material for: Genome-wide TCP transcription factors analysis provides insight into their new functions in seasonal and diurnal growth rhythm in Pinus tabuliformis
Source: BMC Plant Biol. 2022 Apr 2;22:167. doi: 10.1186/s12870-022-03554-4 (PMC8976390; doi:10.1186/s12870-022-03554-4)
Supplement: Supplementary file 8 — Additional file 8. [file 12870_2022_3554_MOESM8_ESM.docx]

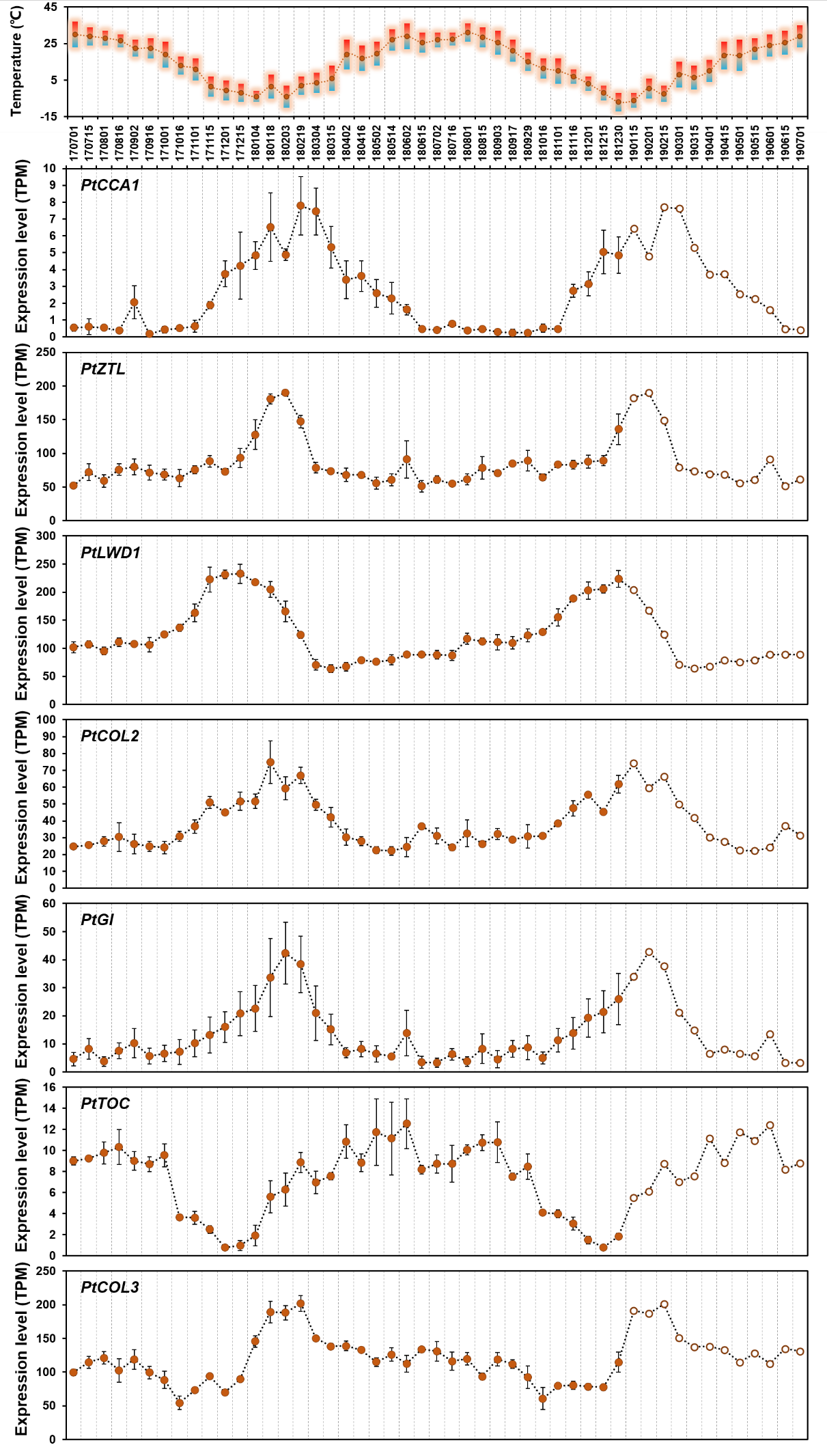


Additional file 8: Fig S3. The expression of *PtCCA1*, *PtZTL*, *PtCOL2*, *PtLWD1*, *PtGI*, *PtTOC* and *PtCOL3*. Monitoring lasts for two years, from July 1，2017 to July 1，2019. Error bars represented variability of three independent replicates.
