# Supplementary material for: Genome-wide TCP transcription factors analysis provides insight into their new functions in seasonal and diurnal growth rhythm in Pinus tabuliformis
Source: BMC Plant Biol. 2022 Apr 2;22:167. doi: 10.1186/s12870-022-03554-4 (PMC8976390; doi:10.1186/s12870-022-03554-4)
Supplement: Supplementary file 9 — Additional file 9. [file 12870_2022_3554_MOESM9_ESM.docx]

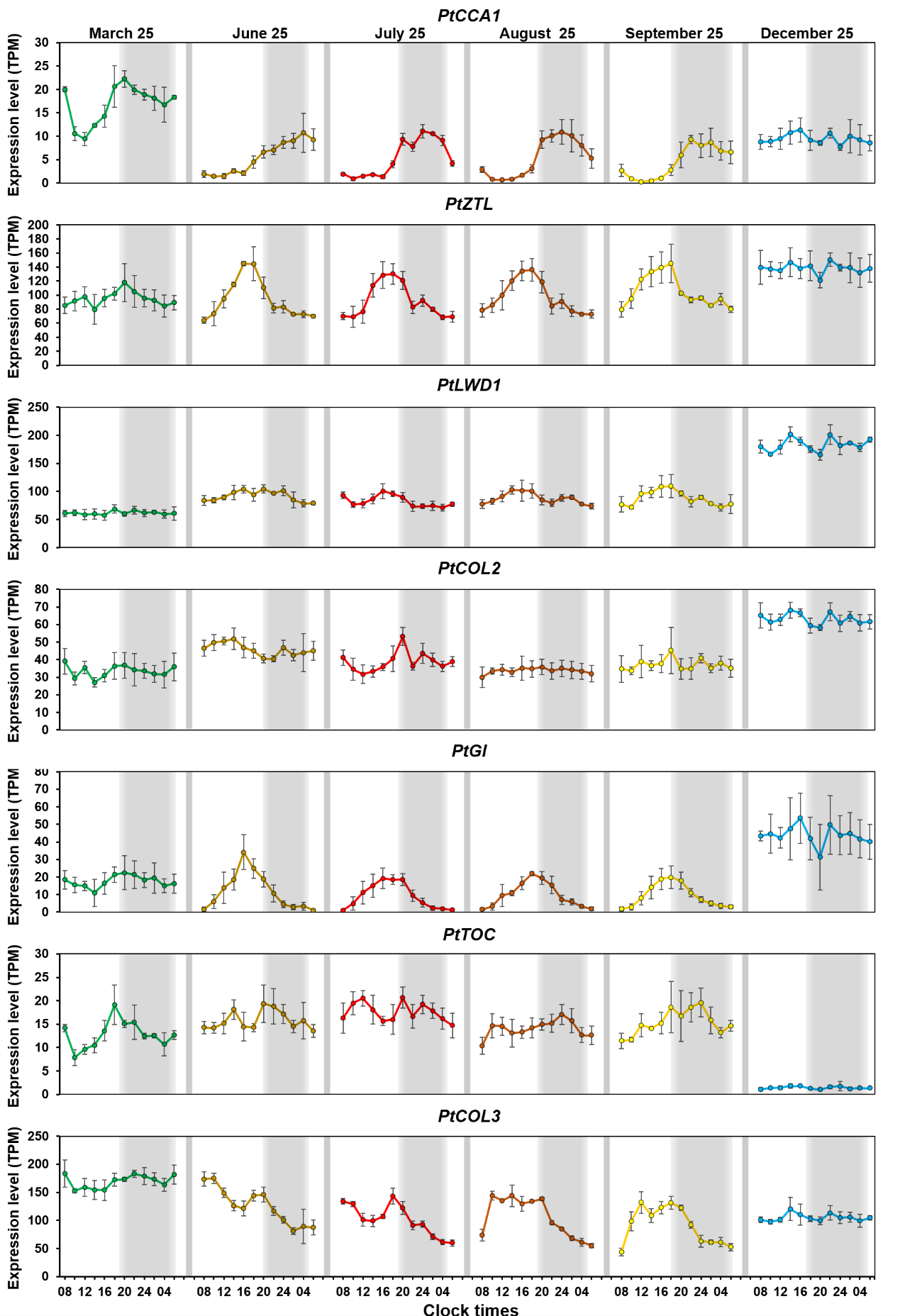


Additional file 9: Fig S4. The expression of *PtCCA1*, *PtZTL*, *PtCOL2*, *PtLWD1*, *PtGI*, *PtTOC* and *PtCOL3* at 8:00, 12:00, 16:00, 20:00, 24:00 and 4:00 in March 25, June 25, July 25, August 25, September 25 and December 25. Error bars represented variability of three independent replicates.
